# Photobiomodulation Therapy Applied after 6 Months for the Management of a Severe Inferior Alveolar Nerve Injury

**DOI:** 10.3390/life11121420

**Published:** 2021-12-17

**Authors:** Marwan El Mobadder, Samir Nammour, Marlin Ortega, Kinga Grzech-Leśniak

**Affiliations:** 1 Dental Surgery Department, Wroclaw Medical University, 50-425 Wroclaw, Poland; kinga.grzech-lesniak@umw.edu.pl; 2 Department of Dental Sciences, Faculty of Medicine, University of Liege, 4000 Liege, Belgium; S.Namour@ulg.ac.be; 3 Physician Associate Pllc, Private Practice, Houston, TX 77030, USA; marlinortegaresearch@gmail.com

**Keywords:** low level laser therapy, dental extraction complications, nerve damage, laser therapy

## Abstract

Despite its significant negative impact on the quality of life, the methods for the management of the inferior alveolar nerve (IAN) injury are still limited. In this case report, the patient did not show any improvement from the day of the iatrogenic accident until 6 months. A significant improvement of the symptoms started to appear only at 6 months when PBMT was applied. A total of 42 sessions of PBMT took place. The application zone included intraoral and extraoral areas. The parameters were: Delivery power of 0.1 W, for 40 s, continuous wave (CW), contact mode, and delivered energy of 4 J. The delivered energy density related to the fiber diameter was 1415 J/cm^2^. Each treated point was considered to be 1 cm^2^ of diameter. At the end of the treatment, all of the symptoms disappeared except for an abnormal sensation on touching the mucosa and gingiva of the concerned area. No side effects were noted. This case report shows that PBMT can be a very promising approach for the management of severe cases that are not improving with conventional methods.

## 1. Introduction

The incidence of iatrogenic and traumatic peripheral nerve injuries is estimated to be about 300,000 cases per year [1,2]. In oral and maxillofacial surgery, the most common nerve injury is the inferior alveolar nerve (IAN) damage during an extraction of the mandibular wisdom tooth. Studies suggest that in 13.2% of mandibular wisdom tooth extractions, inferior alveolar nerve damage occurs and among the 13.2% of cases, only 0.12% persist permanently [3]. The relatively high occurrence of iatrogenic nerve damage in both the medical and dental fields, has led the healthcare community to find effective and reliable solutions that might improve the recovery of the injured nerve and reduce the possible related complications, such as muscle atrophy associated with nerve injury, pain, numbness, motor activity, etc. [4,5,6].

With the current available therapeutic options, it is difficult to restore function and/or sensation in the long-term peripheral nerve injury, which in the case of IAN negatively affects the quality of life and the psychological status of the patient [7,8,9,10,11]. Surgical interventions, such as nerve transfer, nerve conduits, direct nerve repair, fibrin glue, etc. present serious drawbacks with adverse effects that cannot be neglected. In addition, these methods are quite expensive and demand technical expertise [12]. On the other hand, non-surgical methods are still showing limited effectiveness and benefits. These non-surgical approaches include the use of medications, such as analgesics, tropical treatment, corticosteroids, as well as the use of phytochemicals, such as aminopyridine, etc. [12].

Among these approaches, photobiomodulation therapy (PBMT) has shown promising results in several clinical and in vitro studies [13]. Photobiomodulation therapy is defined as a non-thermal therapeutic use of light [14,15]. Today, a robust data suggests that PBMT can be effective in several complications, such as the chronic inflammatory process, oral mucositis, muscle recovery, pain management, etc. It is well-documented that PBMT stimulates the cytochrome C oxidase (CCO), a respiratory energy-transducing enzyme [15,16,17]. This stimulation of CCO will stimulate the mitochondrial activity, which will stimulate the ATP production [15,16,17,18]. In addition, PBMT causes a short, reversible burst of reactive oxygen species (ROS) that is followed by an adaptive reduction in oxidative stress. This will attenuate the inflammatory process [19]. Other mechanisms were also investigated, such as the direct effect that PBMT might have on the neuronal function and on the sensation of pain. Therefore, the exact mechanism of action is believed to be more complex and is still not fully elucidated [20]. A thorough review of literature revealed that in more than 80% of the experimental studies conducted on PBMT for peripheral nerve repair damage, a positive outcome on post traumatic and postoperative nerve recovery was observed [13]. However, studies on the use of PBMT for severe alveolar nerve damage are still poor [13].

In this case report, the patient was referred after 6 months of the injury when all of the other non-surgical methods failed to improve the symptoms. Therefore, the aim of this case report is to illustrate the relatively innovative PBMT treatment protocol and parameters that led to a successful management of severe alveolar nerve damage post-surgical extraction of a mandibular wisdom tooth.

## 2. Case Report

A 28-year-old male, non-smoker patient with no systematic condition, was referred to the clinic complaining of severe symptoms from a post-surgical extraction of the right mandibular wisdom tooth. The consultation was conducted exactly 6 months and 9 days after extraction due to the non-improvement of the symptoms and his refusal to undergo any surgical intervention. The patient, took on a daily basis from week 1 until week 4 post-extraction (21 days exactly), a capsule of 400 UI vitamin D_3_ (Cholecalciferol) (Euro-D, Euro-Pharm International Canada Inc.), Vitamin B1 (as thiamine mononitrate, 100 mg), Vitamin B6 (as pyridoxine HCl, 200 mg), and Vitamin B12 (cyanocobalamin, 200 mcg) (NEUROMED Inc. company, San Clemente, CA 92673, United States). In addition, the patient was instructed to take Prednisolone 50 mg (Mediphar, laboratoires, Beirut, Lebanon) for 10 days post-extraction. After that period, the patient claimed that the symptoms did not improve at all and refused to undergo any surgical intervention. The symptoms and their sites according to the patient were (baseline):

- Complete numbness of the gingiva and the mucosa from the extracted wisdom tooth until the right mandibular canine.
- Robust contraction of the inferior lip.
- Deep cutaneous pain on the inferior lip.
- Complete numbness of more than half of the right chin.
- Generalized and vague pain in the right mandibular arch.

A cold test was conducted from the second mandibular right molar and until the second mandibular left molar. The cold test was conducted with a cold spray applied to a Q-tip and held on a tooth for 5 to 10 s. The cold test revealed a mean value of 3 s delay from the time the stimulus took place and until the sensation. The pain was stronger when compared to the other mandibular arch (from the left incisor to the left second molar), which showed a normal value of pain, within a normal time. Moreover, a cone-beam computed tomography (CBCT) was conducted. Nothing unusual or pathological was detected on the CBCT.

### 2.1. Diagnosis

The diagnosis was a severe iatrogenic alveolar nerve damage during the extraction of the right mandibular wisdom tooth. Based on the medical research council (MRC), the sensory assessment of varying complexity was classified. The mandibular right gingiva and the cheek were classified as S0 which refers to “no sensation at all”. The lower lip was classified as S2 which refers to “deep cutaneous pain in an autonomous zone”. The exact diagnosis between neuropraxia, axonotmesis or neurotmesis was not possible since no surgical intervention or biopsy took place (Table 1, Table 2 and Table 3). Moreover, the patient was diagnosed with Grade IV according to Sunderland’s classification [12,21].

### 2.2. Treatment

**Figure 1:**
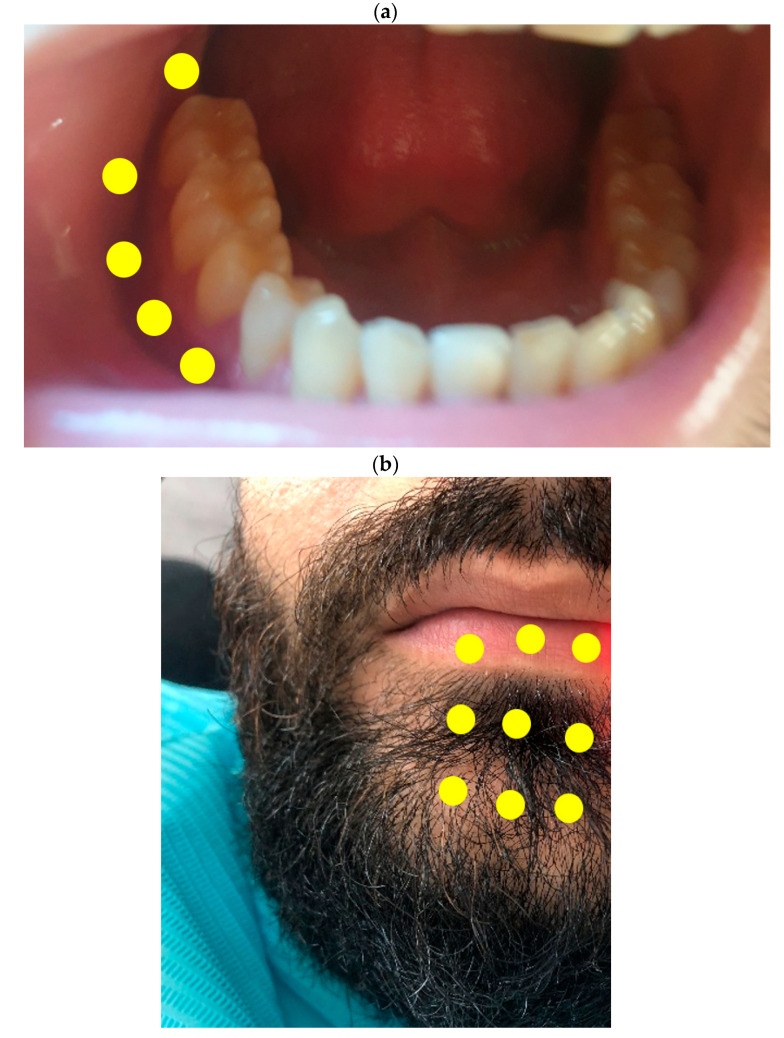
Treatment area of PBMT. The yellow points indicate the areas that were irradiated. (**a**) Intraoral PBMT; 5 of the 6 points are illustrated, the sixth point is on the apical aspect of the canine; (**b**) extraoral PBMT.

Before the treatment, the patient signed a written informed consent. The limitations and possible advantages of the treatment were explained. In addition, the patient was invited to read some related literature, in order to better understand the benefits of PBMT and the recovery after peripheral nerve damage (Figure 1). The treatment protocol consisted of three sessions each week for 2 weeks (this was considered as one series). Therefore, in total, each series was composed of six sessions in 2 weeks. Between one session and another, a 48 h break took place. The symptoms were assessed after each series (six sessions). In total, 42 sessions took place (seven series of six sessions each). After each series, the symptoms were assessed and only if there was an improvement, a new series will start. Concerning the PBMT protocol and parameters, intraoral and extraoral irradiation took place using a diode laser with a wavelength of 635 nm (Smart M, Lasotronix, Warsaw, Poland). Six points were irradiated intraorally (Figure 1a). The points included the vestibular aspect of the cervical area of the gingiva from the second molar to the canine, with the aim to irradiate the trajectory of the inferior alveolar nerve. In addition, care was taken to irradiate the gingiva above the mental foramen where the mental nerve is located. For the extraoral irradiation, nine areas were irradiated on the lower lip and the cheek, in order to target the mental nerve (Figure 1b). Regarding the parameters, the delivered power of 0.1 W was used during 40 s in a continuous wave (CW), corresponding to a delivered energy of 4 J. The delivered energy density related to the fiber diameter was 1415 J/cm^2^. Each treated point was considered to be 1 cm^2^ of diameter (Table 4).

### 2.3. Symptoms Assessment during the Treatment

The symptoms of the patient were progressively improving after each series. At the end of the treatment, which consisted of 42 sessions, a slight abnormal sensation persisted when touching the gingiva and mucosa. The contraction of the lip and cheek disappeared completely. In addition, the cold test gave normal values within a normal range of time between the stimulus and the sensation. Of note, the contraction of the lip and cheek was the last symptom to be treated. The patient was satisfied after the disappearance of the contraction, which was affecting his quality of life and making him always concerned and uncomfortable. After the non-improvement of the gingiva and mucosa on touching after 42 sessions, the treatment was considered complete. Therefore, according to the MRC classification, the lip and cheek had a classification of S4 at the end of the treatment, revealing a normal sensation, and the gingiva and mucosa had a classification of S3, revealing an unnormal sensation only on touching with good stimulus localization (Table 5).

## 3. Discussion

In this case report, the patient was referred 6 months after the iatrogenic inferior alveolar nerve (IAN) damage due to the persistence of the symptoms. The intake of vitamin D, B1, B6, and B12 was not improving the case. In addition, it is well-documented that if the symptoms persist after 6 months of the trauma, the possibility of healing is very unlikely [13]. Interestingly, in this case report, the first signs of improvement were noted after 6 months of the injury and only when PBMT was initiated. Therefore, after 42 sessions, the only symptom that persisted was the abnormal sensation on touching the gingival and mucosal tissue of the right mandibular arch. These findings confirm the effectiveness of PBMT in the management of the IAN cases. Moreover, it was noted that when a break of 10 days took place between each consecutive series of six sessions, there was no improvement of the symptoms. This additional finding confirms that when PBMT is applied, a biostimulation of the healing process is stimulated. IAN dysesthesia is reported to be between 0.35% to 8% and a permanent impairment lasting more than 6 months is reported to be in 0.25–0.91% of the cases [3,22,23,24]. Despite the deleterious impact on the quality of life that the IAN injury has, the treatment approaches are still somehow limited and the results are often aleatory [22,23,24]. In fact, the process of recovery for a peripheral nerve-injury, with or without surgical intervention, is often incomplete [13]. In the majority of cases, patients remain with a loss of sensory which results in severe social and psychological consequences [25]. Therefore, this case report illustrates a very promising approach among the very few available approaches that might be used in order to manage the severe and/or permanent cases of peripheral nerve injury.

The findings of this case report can be explained by understanding the underlying mechanism of action of PBM on both the peripheral nervous system and the muscle. In the literature, it is established that a stimulation of the migration and fiber sprouting of neuronal cell aggregates can be obtained after an adequate application of PBMT. In addition, the enhancement of the development of large-sized neurons with a dense, branched interconnected network of neuronal fibers is also well-demonstrated [26,27,28]. Moreover, experimental peripheral nerve crush injury studies investigating the benefits of PBMT on the nerve injury model in rats showed that PBMT can directly increase the functional activity of the injured peripheral nerve [13,28]; preserve the functional activity of the injured nerve over time; decrease or prevent the scar tissue formulated at the site of injury; and prevent or reduce the degeneration in the corresponding motor neurons of the spinal cord [29]. These results suggest that laser phototherapy accelerates and improves the regeneration of the injured peripheral nerve. On the other hand, the management of the contracted muscles that was obtained in this case report, can be explained by the findings of a study published by Mira M. Mandelbaum-Livnat et al. [13]. In their study, the PBMT therapy increased the biochemical activity and improved the morphological recovery in the muscle, and thus, had direct therapeutic applications on the muscle, especially during progressive atrophy resulting from peripheral nerve injury [13]. Therefore, it seems that PBMT results in a biochemical cascade that occurs not only on the nerve damage, but also on the affected muscles. This beneficial effect on the muscles might be due to an increased activity of creatinine kinase, thus preserving a reservoir of high-energy phosphate that is available for a quick resynthesizing of ATP. This would enable the survival of anti–acetylcholine receptor antibodies that can cause the loss of muscle function by several mechanisms. Moreover, it has been demonstrated that PBMT can significantly decrease the reactive oxygen species formation and the oxidative damage markers, that are generally seen in traumatic muscle injuries [29]. Regarding the improvement of the neuronal activity, the exact biochemical mechanism remains not fully understood. However, PBMT accelerates revascularization and angiogenesis, which leads to a better and/or faster healing process of the injured zone [30]. In addition, PBMT could regulate the nerve growth factor (NGF), which is required for the survival of the developing sympathetic and sensory neurons, especially when the nerve fiber is injured and the neurons need to be protected from apoptosis [31]. Therefore, further research is needed in the future to study these factors.

## 4. Conclusions

The use of photobiomodulation therapy within the suggested protocol and parameters in this case report, showed a significant improvement but not a total recovery of the severe symptoms related to the inferior alveolar nerve injury. The recovery started only when PBMT was applied at 6 months post-injury.

## Figures and Tables

**Table 1:** Sunderland classification of peripheral nerve injuries [12].

Sunderland Classification	Causes	Recovery	Pathophysiology	Surgical Intercessions
Grade IV	Nerve crush	Incomplete and variable—depending on the injury and treatment—months to years	Axon with myelin sheath, endoneurium disconnected	Typically required; procedure depends upon findings

**Table 2:** Medical research council classification of the symptoms in the right mandibular arch.

Area	Classification	Description
Gingiva and mucosa from the second molar to the canine	S0	No sensation at all
Cheek	S0	No sensation at all
Chin	S0	No sensation at all
Lower lip	S1	Deep cutaneous pain in an autonomous zone

**Table 3:** Assessment of the related symptoms.

Area	Description
Teeth from the second right molar to the right canine	Cold test result: A mean value delay of 3.2 s between the stimulus and the sensation of pain
Right depressor labii inferioris muscle	Sensation of contraction with normal function
Right depressor anguli oris	
Right modiolus	
Right mentalis	

**Table 4:** Parameters of the photobiomodulation therapy used per point of irradiation.

Parameters	Values
Wavelength	635 nanometers
Output Power	0.1 Watt
Fiber diameter	600 µm (0.6 mm)
Mode of irradiation	Continuous and contact mode
Time of irradiation	40 s
Energy delivered	4 Joules
Delivered energy related to fiber diameter	1415 J/cm^2^
Number of sessions in total	42 sessions
Irradiation area	Intraorally and extraorally (Figure 1)

**Table 5:** Symptoms in the concerned area on different times of follow-up.

Symptoms in the Concerned Area	Follow-Up Period after Each Session							
	Baseline	After S1	After S2	After S3	After S4	After S5	After S6	After S7
Gingiva and mucosa from the second molar to the canine	No sensation at all	Abnormal sensation with pain on touching					Light abnormal sensation on touching	
Cheek	No sensation at all		Abnormal sensation with deep cutaneous pain	Normal sensation				
Lower lip	Deep cutaneous pain in an autonomous zone		Abnormal sensation with deep cutaneous pain			Abnormal sensation only on touching		Normal sensation
Cold Test on the tooth	3 s delay with strong pain			Normal cold test				
Muscle contraction of the mental region (chin)	Severe contraction			Moderate contraction			No contraction	
Muscle contraction of the lip	Severe sensation of contraction		Moderate sensation of contraction				Light sensation of contraction	No contraction

S: Series (each six sessions were considered as a series).

## Data Availability

Data supporting this case report can be requested from the corresponding author with a reasonable reason.
